# Reporting a novel growth hormone receptor gene variant in an Iranian consanguineous pedigree with Laron syndrome: a case report

**DOI:** 10.1186/s12902-023-01388-1

**Published:** 2023-07-20

**Authors:** Fatemeh Bitarafan, Mehrnoosh Khodaeian, Fatemeh Garrousi, Raziyeh Khalesi, Donya Ghazi Nader, Behnam Karimi, Reza Alibakhshi, Masoud Garshasbi

**Affiliations:** 1 Department of Medical Genetics, DeNA Laboratory, Tehran, Iran; 2 Department of Medical Genetics, Oslo University Hospital, Oslo, Norway; 3 Medical Genetics Laboratory of Dr. Alibakhshi, Sobhan Medical Complex, Kermanshah, Iran; 4 Department of Biochemistry, School of Medicine, Kermanshah University of Medical Sciences, Kermanshah, Iran; 5 Department of Medical Genetics, Faculty of Medical Sciences, Tarbiat Modares University, Tehran, Iran

**Keywords:** Laron Syndrome (LS), *GHR*, Whole Exome Sequencing (WES), Iranian population, Case report

## Abstract

**Background:**

Human growth hormone (hGH) plays a crucial role in growth by binding to growth hormone receptor (GHR) in target cells. Binding of GH molecules to their cognate receptors triggers downstream signaling pathways leading to the transcription of several genes, including insulin-like growth factor (IGF)-1. Pathogenic variants in the *GHR* gene can result in structural and functional defects in the GHR protein, leading to Laron Syndrome (LS) with the primary clinical manifestation of short stature. So far, around 100 *GHR* variants have been reported, mostly biallelic, as causing LS.

**Case presentation:**

We report on three siblings from an Iranian consanguineous family who presented with dwarfism. Whole-exome sequencing (WES) was performed on the proband, revealing a novel homozygous missense variant in the *GHR* gene (NM_000163.5; c.610 T > A, p.(Trp204Arg)) classified as a likely pathogenic variant according to the recommendation of the American College of Medical Genetics (ACMG). Co-segregation analysis was investigated using Sanger sequencing.

**Conclusions:**

To date, approximately 400–500 LS cases with *GHR* biallelic variants, out of them 10 patients originating from Iran, have been described in the literature. Given the high rate of consanguineous marriages in the Iranian population, the frequency of LS is expected to be higher, which might be explained by undiagnosed cases. Early diagnosis of LS is very important, as treatment is available for this condition.

**Supplementary Information:**

The online version contains supplementary material available at 10.1186/s12902-023-01388-1.

## Background

Laron syndrome (LS), also known as Growth Hormone Insensitivity (GHI), (OMIM: 262,500), is a condition characterized by a failure to respond to both endogenous or exogenous growth hormone (GH), resulting in short stature [70]. LS occurs in approximately 1 in 1,000,000 babies born each year [39] and is caused by a defect in the *GHR* gene. The main phenotypic feature of LS is dwarfism, which can be observed in infants, older children or adults. Other symptoms of LS include atypical facial features, such as a protruding forehead, saddle nose, puffy cheeks, small hands and feet, obesity, delayed bone age, small genitalia in boys, decreased bone mineral density with a greater risk of osteoporosis, lipid disorders, cardiovascular disease, hypothyroidism, chronic kidney disease, and malnutrition [16, 43, 59].

Biallelic variants in the *GHR* are the most prevalent causes for primary GHI syndrome with classical GHI, phenotypically presenting with severe short stature and prominent insulin-like growth factor (IGF)-1 deficiency. Recently, milder GHI syndrome has been reported to be caused by rare monoallelic *GHR* mutations that can lead to a dominant-negative effect [49, 59, 69].

Biochemically, it has been reported that patients with LS have very low serum levels of IGF-1, IGF binding protein (IGFBP)-3, GH binding protein (GHBP), and acid-labile subunit (ALS) while they show increased secretion of GH [7, 59].

Dwarfism is mostly caused by mutations in the *GHR* gene which is characterized by normal or elevated GH levels in serum and low levels of IGF [44]. GH has a wide effect on the growth of skeletal and soft tissues and by binding to the growth hormone receptor (GHR), it has a metabolic effect on target cells, which is the first step in the action of GH [16].

Human GH belongs to a family of polypeptides which is synthesized by anterior pituitary gland cells and stimulates growth [48]. GHR is a homodimeric cytokine receptor that lacks intrinsic kinase activity and recruits Janus kinase 2 (JAK2) for activation of downstream signaling pathways, resulting in the transcription regulation of target genes, including IGF-1 [59, 69].

The human GHR cDNA encodes 638 amino acids [14] and is located on chromosome 5p13.1–12, which includes 9 introns and 10 exons [43]. Exons 2 to 7 encode the extracellular domain (ECD) of the receptor, including 246 amino acids, exon 8 encodes the transmembrane domain, including 24 amino acids, and exons 9 and 10 encode the cytoplasmic intracellular domain (ICD), including 350 amino acids [32, 51].

Today around 100 variants have been identified in the *GHR* gene that can cause dwarfism in humans, including missense, deletion, nonsense, frameshift and splice site variants. These variants affect signal transduction, ligand binding, and GHR dimerization [44, 49]. Studies have shown that recombinant IGF-1 therapy is the only effective treatment and can improve height z-score in LS cases compared to untreated patients, although standard height may not be achieved. Several factors may affect treatment response, including the age of the children, poor treatment compliance, and their genotype. Case studies reporting treatment results can be helpful in identifying association between variants and treatment response. However, as the disease is rare, it is not feasible to investigate the association between LS variants and treatment response [53].

In this report, we describe an unreported, likely pathogenic variant p.(Trp204Arg) in the ECD of GHR leading to LS in 3 siblings born to a consanguineous family.

## Case presentation

### Clinical findings

A 32-year-old man and his two siblings from Kermanshah, Iran, who were suspected to have LS, were referred to us. The parents were consanguineous and the suspected siblings exhibited childhood-onset short stature.

All available family members provided written informed consent. This project was conducted ethically in accordance with the World Medical Association Declaration of Helsinki.

**Fig. 1:**
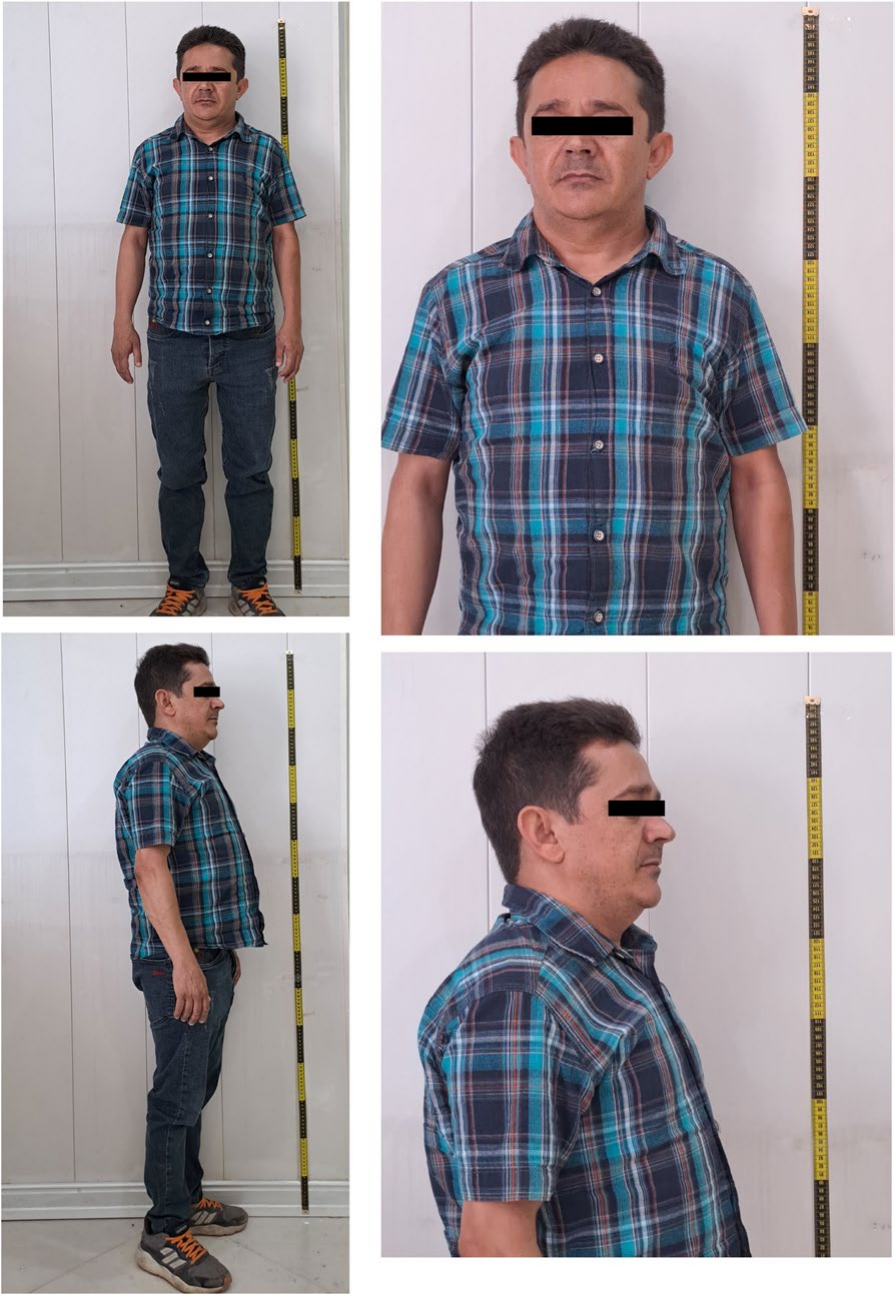
Photographs of the proband at the age of 32 years

The 32-year-old proband exhibited a delay in skeletal maturity of about 13 years. He was 146 cm tall [height,—3.3 Standard Deviation Score (SDS)] with a small chin, double chin, and a mildly prominent forehead (Fig. 1). He had a history of developing kidney stones and had undergone surgery for approximately four episodes of kidney stones at the age of 7, 10, 14, and 20.

The patient’s mother was 145 cm (-1.7 SDS) and had high blood pressure and kidney stones. The father was 155 cm tall (-2.1 SDS) and also had hypothyroidism, kidney stones that were operated when he was 65, and had a history of stroke. The patient had another affected brother and sister, whose heights were 136 (-4.6 SDS) and 138.5 (-2.6 SDS) cm, respectively (Supplementary Fig. 1). They also exhibited delayed bone age, small chin, double chin, and a mildly prominent forehead. The affected sister also suffered from kidney stones, and her neonatal head circumference, height, and weight were below the normal range. Three other brothers were healthy, and their heights were in the normal range.

All affected individuals were born at term, and their pregnancies and deliveries were normal. The parents stated that hypoglycemia was never noted in the affected individuals, although it was not documented. The developmental milestones of all affected individuals were normal, and no special abnormalities were reported during their childhood. The affected siblings claimed that they received hormonal therapy in childhood, although there are no records available.

The proband experienced delayed puberty at the age of 17 years. His semen analysis report at the age of 31 showed normal sperm count, motility, progressive motility, sperm morphology, semen liquefaction time and semen PH (Table 1). He had no fertility problems.

**Table 1:** Semen analysis report of patient

**Main Parameters**	Result	Status	Normal Values
Semen volume (ml)	2.00 ml	Normal	≥ 2
Semen pH	7.50	Normal	7.2–8
Sperm Density (million/ml)	28.47	Normal	More than 20
Motile sperm (%)	79.04	Normal	More than 60
Progressive Sperm (%)	72.93	Normal	A ≥ 25% or A + B ≥ 50%
Normal Morphology (%)	65	Normal	More than 40
**Speed Assessment**			
Class		Percent	
A (Rapid Progressive)		45.41	
B (Slow Progressive)		27.51	
C (Non Progressive)		6.11	
D (Immotile)		20.96	

### Molecular findings

A High Pure PCR template preparation kit (Roche; Product No, 11,814,770,001) was used to extract genomic DNA from the patient and all available family members for WES.

The WES was performed on 1 μg of genomic DNA sample from the proband according to the latest reference genome assembly GRCh38/hg38 (https://genome.ucsc.edu/GRCh38/hg38) using Agilent's SureSelect Human All Exon V6 kit. To obtain an average coverage depth of approximately 100 × , the generated library was sequenced on a HiSeq 4000 Illumina platform. Heterozygous variants were excluded considering the homozygous inheritance mode in the pedigree. Consequently, variants with allele frequencies more than 1% were excluded according to dbSNP132 (https://www.ncbi. nlm.nih.gov/projects/SNP), the 1000 Genomes Project [63], the Exome Sequencing Project (ESP) (http://evs.gs.washi ngton.edu/ EVS), the Exome Aggregation Consortium (ExAC), GnomAD [35], and the local database, Iranome [20]. Intragenic variants, untranslated regions (UTRs), and synonymous variants were also excluded. Pathogenic variants reported in the Human Gene Mutation (HGMD) [66] and ClinVar [42] were given a higher priority.

To predict the pathogenic consequences of the detected variants, SIFT [52], PolyPhen-2 [1], and MutationTaster [62] databases were used. The WES analysis identified a homozygote variant (c.610 T > A) in the *GHR* gene (NM_000163.5) on chromosome 5.

**Fig. 2:**
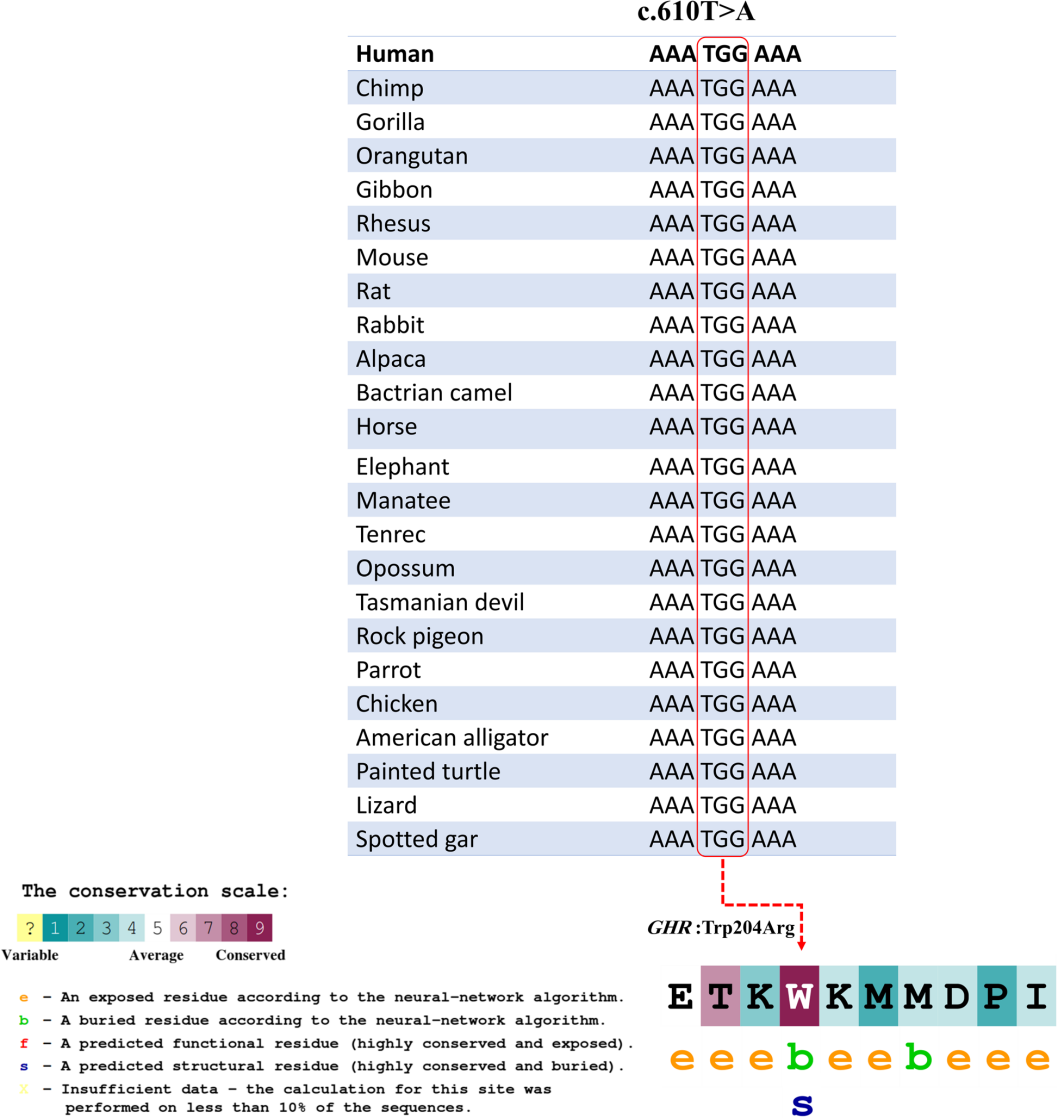
UCSC database was used to show the multiple sequence alignment displaying evolutionary conservation of c.610 T nucleotide and Trp204 (W) in the *GHR* gene among different species. Meanwhile, the amino acid sequence of the GHR protein is colored according to the conservation scores provided by the ConSurf server

To check the evolutionary conservation for the region of the variant, ConSurf (http://www.consurf.tau.ac.il) and UCSC database [40] were applied (Fig. 2). Multiple alignments of the amino acid sequence with various species had revealed that the Trp at position 204 in human GHR is evolutionarily conserved across species, including Primates, Euarchontoglires, Laurasiatheria, Afrotheria, Mammals, Birds, Sarcopterygii, and Fish.

To validate the candidate variant, Sanger sequencing was performed first on the proband and subsequently on all available family members to confirm co-segregation of the selected variant with LS (Supplementary Fig. 1).

The web-based server Primer3Plus (https://primer3plus.com/cgi-bin/dev/primer3plus.cgi) was used to design primers. The in-silico PCR tool in the UCSC genome browser (https://genome.ucsc.edu/cgi-bin/hgPcr) and dbSNP database (https://www.ncbi.nlm.nih.gov/snp) were utilized to check the specificity of primers and lack of SNPs in the genomic region, respectively. PCR products were sequenced by an ABI 3130 system using ABI PRISM BigDye Terminator v. 3.1 sequencing kit (Applied Biosystems, USA), and the sequencing data were analyzed using CodonCode Aligner v. 8.0.2 (CodonCode Corp, USA). Primers and PCR conditions are available upon request.

Sanger Sequencing results confirmed the co-segregation of the variant with the disease, as both parents showed a heterozygote pattern of the variant. The three affected siblings were homozygous mutant, while the 3 healthy siblings were heterozygote for the c.610 T > A variant.

According to the recommendations of the ACMG for the classification of sequence variants [57], this variant is classified as likely pathogenic due to the following evidence: 1. The variant is located in the ECD, which is a mutational hot spot of the GHR and also in the FN3 domain,a vital and well-established functional domain (PM1), 2. The variant was absent in control population databases, including the 1000 Genomes Project, Exome Aggregation Consortium, and Exome Sequencing Project (PM2), 3. Missense variants are a common mechanism of the LS syndrome, and the *GHR* gene has a low rate of benign missense variation (PP2), 4. Various lines of computational data including 10 pathogenic predictions from BayesDel_addAF, DEOGEN2, EIGEN, FATHMM-MKL, M-CAP, MVP, MutationAssessor, MutationTaster, REVEL, and SIFT suggest a damaging effect on the gene, as the variant is located in a highly conserved region, FN3 domain, which is considered a supporting evidence for pathogenicity (PP3), 5. The patient’s phenotypes are highly similar to LS cases (PP4).

**Fig. 3:**
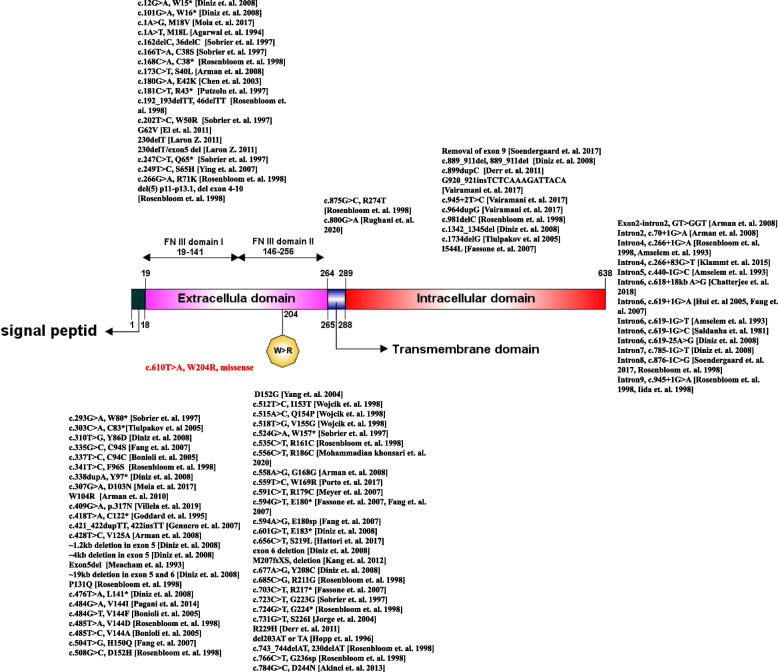
Schematic representation of reported variants in the *GHR* gene and the corresponding protein structure including the signal peptide, an extracellular domain consisting of two FN3 subdomains, a transmembrane domain, and an intracellular domain. The identified variant in this study was marked in red [2–6, 8, 12, 13, 15, 17–19, 21, 23, 26–29, 31, 34, 36, 37, 43, 47, 48, 50, 51, 54–56, 58–60, 64, 65, 68–70, 72–74]

Using IBS illustrator [46], a schematic pattern of GHR protein, its domains, and the reported variants related to LS has been shown in Fig. 3.

## Discussion and conclusion

Binding of GH to GHR triggers a cascade of downstream signaling pathways, including IGF-1 production, which enables GH to play a prominent role in growth [69]. Pathogenic variants in the *GHR* gene can result in IGF-1 deficiency and impaired binding affinity of GHR to GH, leading to reduced levels of related molecules such as IGFBP-3 [30, 39, 69].

To date, about 400 to 500 LS patients with mutations in the human *GHR* gene have been reported [71]. The condition arises from biallelic and occasionally monoallelic variants in the *GHR* gene [59]. Biallelic variants typically result in a classical form of GHI, which is characterized by severe short stature, IGF-1 deficiency, obesity, delayed bone age, and facial dimorphisms. In contrast, monoallelic variants may present with milder clinical features and may be identified as having idiopathic or familial short stature, or may remain undiagnosed [50, 59, 69]. In cases with heterozygous status, compensatory mechanisms that remain unidentified may enable them to achieve a normal height range, which is insufficient in homozygous or compound heterozygous cases [51].

Most reported cases with biallelic variants belong to ethnic groups where consanguineous marriages are prevalent due to various factors such as tradition, culture, and religion, including two large groups living in Ecuador and Israel [43, 50, 69]. Consanguineous marriages have long been recognized as a significant etiological factor in the prevalence of recessive genetic disorders [25].

Laron et al. reported an Israeli cohort comprising 9 Jewish-Iranian, 1 Jewish-Iranian/Turkish, and 1 Moslem-Iranian families [43]. Among six of these families, four homozygous deletion variants were identified. Three families carried deletions in exon 5 and 6 and one family carried a 230delT variant. In the Jewish-Iranian/Turkish family, compound heterozygous 230delT/exon 5 del was identified, while in the Moslem-Iranian family, a point mutation, Y86D in exon 5, was reported [43]. No additional homozygous variants leading to Laron syndrome have been reported in Iranian population since then. A heterozygous mutation c.556C > T,p.R186C in exon 6 has also been reported in an Iranian family with short stature [50].

Several variants in the *GHR* gene affecting ligand binding, GHR dimerization, or signal transduction have been reported, and most of these variants are located in the extracellular domain of the receptor [44]. The ECD is encoded by exons 2–7 and comprises 246 amino acids, which are organized into two functional subdomains: subdomain 1 (residues 19–141) is involved in GH binding, and subdomain 2 (residues 146–264) is responsible for receptor dimerization and GH-induced receptor rotation [44]. Two fibronectin type III (FN3) subdomains, each consisting of 7 antiparallel β-strands, are located within the ECD domain. The first one spans residues 19–141, while the second one consists of residues 146–256, which are linked by four amino acids [32]. The FN3 domain is a small, autonomously folding unit that occurs in approximately 2% of all animal proteins involved in ligand binding [38]. During cellular signaling pathways, the GH molecule binds to the FN3 subdomains of GHR, leading to their conformational alteration and activation [32].

In this study, we identified a novel missense variant, W204R, in a highly conserved residue of the GHR protein in the proband. Subsequent screening of other available family members revealed its presence in two other affected siblings.

The parents and 3 healthy brothers were found to be heterozygous. In line with previous literature, heterozygous family members including the parents and three healthy brothers showed different heights, suggesting the role of other variants [50, 51]. Height is a complex polygenic trait controlled by many genes and pathways essential for skeletal growth [16].

In this family, the father’s height was 155 cm (-2.1 SDS), and the mother’s height was 153 cm (-1.7 SDS), while his three heterozygous brothers were 168 (-0.3 SDS), 171 (0.1 SDS), and 174 (0.5 SDS) cm tall.

The growth hormone/insulin-like growth factor-1 (GH/IGF-1) axis plays a critical role in normal human linear growth, and impairment of several genes can lead to GHI. Biallelic mutations in the *GHR*, *STAT5B*, *IGF1*, and *IGFALS* genes are associated with classical GHI. In addition, heterozygous dominant-negative variants in some of these genes including *GHR* and *STAT5B* have been also reported in cases presented with milder phenotypes [61, 67].

Mutations in *STAT5B* are associated with severe growth failure, followed by IGF-1 deficiency, and insensitivity to GH, accompanied by immunodeficiency. Other complications seen in patients with *STAT5B* mutations include postnatal growth failure, mild face hypoplasia, pubertal delay, and hypoglycemia [41, 61, 67].

In patients with *IGF1* mutations, the prominent complication are intrauterine growth retardation (IUGR), deafness, intellectual and neurodevelopmental delay [41, 61, 67]. In patients with *IGF2* mutations, growth failure and relative microcephaly are the most important features [41, 61, 67].

Like patients with pathogenic variants in *GHR* and *STAT5B* genes, patients with *IGFALS* mutations also experience a pubertal delay. However, *IGFALS* mutations are associated with mild or moderate short stature [61, 67].

*GHR* mutations lead to mild to severe growth failure. Pubertal delay, hypoglycemia, short stature, impaired linear growth, altered skeletal maturation, and elevated GHBP, insulin, and free fatty acid levels are seen in patients with GHR mutations [41, 61]. Patients may also experience craniofacial changes and early childhood obesity [41, 67].

The GH/IGF-1 axis also regulates a wide range of biological functions in several organs such as the kidney. GH/IGF-1 axis disorders are linked to chronic renal failure as it contributes to renal development, electrolyte balance, and body water homeostasis. It also affects glomerular filtration and tubular handling of glucose, sodium, phosphate, and calcium [24, 33]. Studies have shown that the disruption of the *GHR* gene in mouse models result in reduced kidney size. In line with animal study reports, the proband in this study presented with small kidneys size [22, 45]. The proband, his affected sister, and their parents also suffered from kidney stones, a feature not typically observed in Laron patients.

It is known that the GH/IGF-1 system impacts Sertoli cell (SC) proliferation and function as well. Animal model studies have shown that the function of the hypothalamus–pituitary–testicular (HPT) axis can be affected by IGF-1. Studies on Laron’s cases exhibited higher rate of delayed puberty in them. The onset of puberty was noticeably more delayed in Laron boys (15.6 ± 2.6 years) than in girls (11.8 ± 0.2 years). Delayed puberty in males is evident by absence of testicular volume enlargement at 14 years of age. Up to now, no investigation has described sperm parameters or fertility in Laron patients. However, there is no report of reduction in reproductive function in these patients [9–11]. In this study, the proband exhibited a delayed onset of puberty at 17 years of age. At the age of 31, he underwent a semen analysis, which revealed normal sperm count, motility, progressive motility, sperm morphology, semen liquefaction time, and semen pH. He is married and he has no fertility disorders.

The identified variant in this study is located inside exon 6, which encodes part of the protein’s extracellular domain. This mutation replaces a highly conserved polar-neutral tryptophan with a basic amino acid arginine, altering the residue’s charge. Thus it is expected to have a significant impact on the protein’s structure and function.

In summary, we reported a novel biallelic *GHR* variant p.(W204R) associated with LS in three siblings of an Iranian consanguineous family. The observed variation in the height of heterozygote parents and the three healthy brothers is in favor of compensatory mechanisms. To our knowledge, it is the first report of a biallelic *GHR* variant related to LS on Iranian population since the Israeli cohort investigation that included 11 immigrant Iranian families. Accurate identification of causative variants plays a key role in affected families, enabling them to benefit from preimplantation genetic diagnosis (PGD), prenatal diagnosis (PND), or further therapy strategies.

## Supplementary Information

**Additional file 1: Supplementary Figure 1.** Pedigree of the consanguineous LS family and Sanger sequencing chromatograms showing nucleotide sequences of *GHR* in the regions of c.610T>A which is found in the family. Selected patient for exome sequencing is indicated by an arrow. WT/WT: unaffected who were homozygous for wild-type allele; MT/MT which show by green symbols: affected and homozygous for the variant; WT/MT which show by dotted symbols indicate carrier status. Square: male; circle: female; parallel lines: consanguineous marriage.

## Data Availability

The data that support the findings of this study are available on request from the corresponding author.

## Abbreviations

hGH: Human growth hormone
GHR: Growth hormone receptor
IGF: Insulin-like growth factor
LS: Laron Syndrome
WES: Whole Exome Sequencing
ACMG: American College of Medical Genetics
GHI: Growth Hormone Insensitivity
GH: Growth hormone
IGFBP: IGF binding protein
GHBP: GH binding protein
ALS: Acid-labile subunit
JAK2: Janus kinase 2
ECD: Extracellular domain
ICD: Intracellular domain
SDS: Standard Deviation Score
ESP: Exome Sequencing Project
ExAC: Exome Aggregation Consortium
UTRs: Untranslated regions
HGMD: Human Gene Mutation
FN3: Fibronectin type III
GH/IGF-1: Growth hormone/insulin-like growth factor-1
IGFBPs: IGF-binding proteins
IUGR: Intrauterine growth retardation
SC: Sertoli cell
HPT: Hypothalamus–pituitary–testicular
PGD: Preimplantation genetic diagnosis
PND: Prenatal diagnosis
